# Evidence that transport of iron from the lysosome to the cytosol in African trypanosomes is mediated by a mucolipin orthologue

**DOI:** 10.1111/mmi.12285

**Published:** 2013-06-27

**Authors:** Martin C Taylor, Alex P McLatchie, John M Kelly

**Affiliations:** Department of Pathogen Molecular Biology, London School of Hygiene and Tropical MedicineKeppel St., London, WC1E 7HT, UK

## Abstract

Bloodstream-form *Trypanosoma brucei* acquire iron by receptor-mediated endocytosis of host transferrin. However, the mechanism(s) by which iron is then transferred from the lysosome to the cytosol are unresolved. Here, we provide evidence for the involvement of a protein (*Tb*MLP) orthologous to the mammalian endolysosomal cation channel Mucolipin 1. In *T. brucei*, we show that this protein is localized to the single parasite lysosome. *TbMLP* null mutants could only be generated in the presence of an expressed ectopic copy, suggesting that the protein is essential. RNAi-mediated ablation resulted in a growth defect *in vitro* and led to a sevenfold increase in susceptibility to the iron-chelators deferoxamine and salicylhydroxamic acid. Conditional null mutants remained viable when the ectopic copy was repressed, but were hypersensitive to deferoxamine and displayed a growth defect similar to that observed following RNAi. The conditional nulls also retained virulence *in vivo* in the absence of the doxycycline inducer. These data provide strong evidence that *Tb*MLP has a role in import of iron into the cytosol of African trypanosomes. They also indicate that even when expression is greatly reduced, there is sufficient protein, or an alternative mechanism, to provide the parasite with an adequate supply of cytosolic iron.

## Introduction

Iron is the Earth's most abundant metal and is required for the growth and replication of almost all organisms. It is an essential component of many enzymes involved in energy metabolism, as haem or iron-sulphur clusters, due to its versatile redox chemistry. It is also necessary for DNA synthesis as it acts both as a cofactor for ribonucleotide reductase and as part of an iron-sulphur cluster in eukaryotic DNA polymerases (Netz *et al*., 2012). Iron is of such importance to pathogenic microorganisms that mammals have developed an iron sequestration response. This is regulated by the innate immune system through IL-6 or IL-22 induced expression of the peptide hormone hepcidin and acts to minimize iron bioavailability (Wrighting and Andrews, 2006; Armitage *et al*., 2011). Hepcidin-mediated iron deprivation may be crucial for controlling infections with the malaria parasite and a range of bacterial infections (Ratledge, 2004; McDermid and Prentice, 2006; Boelaert *et al*., 2007; Portugal *et al*., 2011). This cytokine-mediated iron limiting response is also associated with the anaemia seen in animal models of trypanosomiasis and in Nagana (animal African trypanosomiasis) (Stijlemans *et al*., 2008; 2010a,b[Bibr b52],[Bibr b53],[Bibr b54]). Anaemia is one of the major causes of death or morbidity resulting from Nagana, an infection of vital economic importance to livestock-farming in Sub-Saharan Africa.

Bloodstream forms of the African trypanosome *Trypanosoma brucei* obtain iron from the host iron-carrier protein transferrin. The parasites express a transferrin receptor in their flagellar pocket (FP), encoded by two closely related genes (*ESAG6* and *ESAG7*) which are transcribed from the variant surface glycoprotein expression site (Chaudhri *et al*., 1994; Ligtenberg *et al*., 1994; Steverding *et al*., 1994; 1995[Bibr b50],[Bibr b51]). Expression of the ESAG 6/7 transferrin receptor can be regulated by iron levels. The mechanism involved has not been identified but differs from the mammalian iron-response system (Fast *et al*., 1999; Mussmann *et al*., 2004; van Luenen *et al*., 2005). The ESAG 6/7 heterodimer is attached to the membrane by a single GPI anchor on the ESAG 6 subunit. On binding of transferrin, the receptor/transferrin complex is endocytosed. Iron is then released in the late endosome/lysosome after acidification, the transferrin is proteolytically degraded by a cathepsin L like enzyme (Steverding *et al*., 2012), and the receptor is recycled back to the flagellar pocket membrane (Kabiri and Steverding, 2000; Pal *et al*., 2003). However, it is unclear how the released iron is subsequently transported into the cytoplasm for utilization by the trypanosome. Iron bound to transferrin is in the Fe^3+^ form, which is practically insoluble (maximal solubility 10^−18 ^M) at physiological pH and temperature. To be exported from the endolysosomal system into the cytoplasm, it must first be reduced to Fe^2+^.

In mammalian cells, the pathway by which iron gets from endosomes to its final destination has still to be definitively resolved. The route may vary between different cell types, depending on their iron requirements (Sheftel *et al*., 2007). The best-studied mechanism for export of iron from mammalian endosomes is the divalent metal transporter DMT1 (also called SLC11A2, NRAMP2). This protein is required for dietary iron uptake in the gut, and for erythropoiesis as well as normal iron homeostasis (Theil, 2011). However, it is not required for iron uptake in all cell types (Gunshin *et al*., 2005). Mucolipin 1 (MCOLN1, TRPML1), a member of the transient receptor potential subfamily of ion channels, can also function as an endosomal iron channel in mammals and may facilitate iron release in cells where DMT1 is not expressed (Dong *et al*., 2008). Mutations within the human *MCOLN1* gene can lead to type IV mucolipidosis (MLIV), a lysosomal storage disease characterized by psychomotor retardation, corneal clouding, retinal degeneration and often iron-deficiency or clinical anaemia (Altarescu *et al*., 2002). MLIV cells have enlarged lysosomes characterized by elevated levels of iron and zinc, as well as lipofuscin (Dong *et al*., 2008; Eichelsdoerfer *et al*., 2010). MCOLN1 is also permeable to Ca^2+^ and is thought to play a role in regulation of membrane trafficking events via PI(3,5)P_2_-mediated activation of Ca^2+^ release (Dong *et al*., 2010). It contains a consensus active-site motif for serine lipase (GYSDG), which is responsible for the membrane remodelling activity of MCOLN1 that regulates the formation of tubulo-vesicular endomembrane structures and lysosomal exocytosis (LaPlante *et al*., 2011). Thus, the mammalian MCOLN1 protein is bifunctional. MCOLN2 and 3 lack the serine lipase domain, but are otherwise conserved, and have not been implicated in mucolipidosis.

DMT1 orthologues appear to be absent from the trypanosomatid genomes, and endosomal iron transport must be mediated by an alternative route. We report here the identification of a trypanosomal orthologue of the MCOLNs, *Tb*MLP (Mucolipin-like protein). The protein is confined to the endomembrane system, and appears to play a major role in iron metabolism within the parasite.

## Results

### Identification of an orthologue of Mucolipin 1 in the trypanosomatid genomes

**Figure 1:**
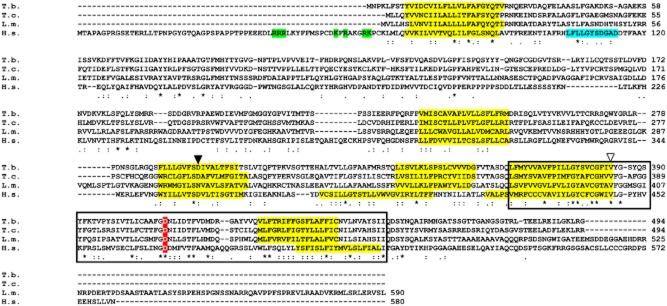
Alignment of trypanosomatid mucolipin orthologues with human MCOLN1. The putative transmembrane regions (yellow), PI(3,5)P_2_ binding (green) or serine lipase active site (cyan) (LaPlante *et al*., 2011) are all highlighted. The boxed region represents the cation channel domain. The white triangle corresponds to a V→L alteration seen in MLIV patient 2 (Altarescu *et al*., 2002), while the black triangle relates to D→Y change that abolishes Fe^2+^ transport in mammalian cells (Dong *et al*., 2008). A transgene carrying the D→K mutation cannot complement MLIV cells (Pryor *et al*., 2006). Identifiers: T.b. – *Trypanosoma brucei* Tb927.7.950, T.c. –*Trypanosoma cruzi* TcCLB.508215.6, L.m. – *Leishmania major* LmjF.26.0990, H.s. – *Homo sapiens* NP_065394.1 (kinetoplastid genes identified with TriTrypDb/GeneDb codes, mammalian proteins with NCBI locus numbers). Asterisks indicate conserved residues. Alignment was carried out using clustalw2 at http://www.ebi.ac.uk/Tools/msa/clustalw2/.

The human MCOLN1 sequence (NP_065394.1) was used to interrogate the trypanosomatid genome data (Hertz-Fowler *et al*., 2004; Berriman *et al*., 2005; El-Sayed *et al*., 2005; Ivens *et al*., 2005; Aslett *et al*., 2010). Orthologous sequences were identified in all the trypanosomatids, including the agents of Chagas disease (*Trypanosoma cruzi*) and the leishmaniases (*Leishmania major, L. infantum*). Intriguingly, in *Leishmania braziliensis*, the orthologue is a pseudogene in which the middle portion of the coding sequence has been deleted (annotated as LbrM.26.1000 http://tritrypdb.org/), although an open reading frame (ORF) is preserved. The extant ORF either is non-functional or may have another role in *L. braziliensis*. The *T. brucei* gene (Tb927.7.950) has an ORF of 1482 bp. The encoded protein has six predicted integral transmembrane domains (identified using PSORT at http://psort.hgc.jp/form2.html), a large luminal loop between transmembrane domains 1 and 2, a putative pore domain between transmembrane domains 5 and 6 and shares similar hydropathy profiles to MCOLN1. Several critical residues in transmembrane helices 5 and 6 and the pore domain, are conserved, including some which are mutated in MLIV patients (Fig. 1, Altarescu *et al*., 2002). Conserved residues were largely restricted to the carboxyl-terminal pore domain encompassing transmembrane domains 5 and 6, with the amino-terminal domain being poorly conserved. The serine lipase active site of MCOLN1 (consensus motif: ([LIV] {KG} [LIVFY] [LIVMST] G [HYWV] S {YAG} G [GSTAC]) (cyan, Fig. 1) is absent from the first luminal loop suggesting that the kinetoplastid orthologue could be a monofunctional cation channel that lacks membrane remodelling capability. The kinetoplastid proteins also lack the amino-terminal cytoplasmic polybasic region implicated in PI(3,5)P_2_ binding in mammalian MCOLN1 (indicated in green, Fig. 1) (Dong *et al*., 2010).

### TbMLP is localized to the endolysosomal system and is concentrated in the lysosome

**Figure 2:**
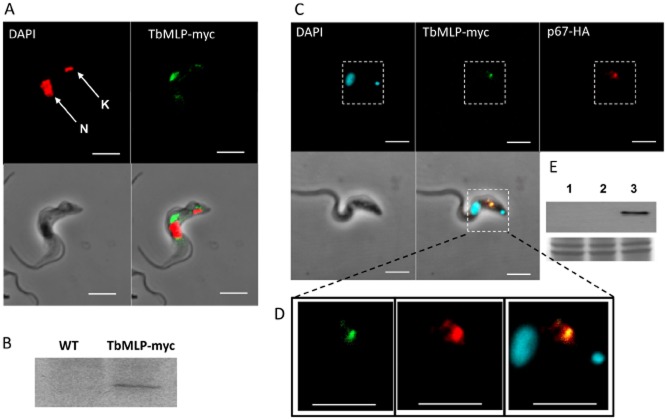
Localization of carboxyl-terminally c-myc-tagged *Tb*MLP (*Tb*MLP-myc) in bloodstream-form trypanosomes.A. *Tb*MLP-myc was localized with monoclonal antibody 9E10 and is shown as green; red indicates the nucleus (N) and kinetoplast (K) stained with DAPI. *Tb*MLP is concentrated in a vesicular structure between nucleus and kinetoplast with some lighter staining around the flagellar pocket. White bar represents 5 μm.B. Western blot to show that antibody 9E10 recognizes a specific band in *Tb*MLP-myc transformed cells; WT indicates wild type.C. *Tb*MLP-myc colocalizes with lysosomal glycoprotein p67. To confirm lysosomal localization of *Tb*MLP-myc the lysosomal marker protein p67 was also tagged with the HA epitope (p67-HA). *Tb*MLP-myc localization is shown in green, p67-HA in red and DAPI stained DNA in cyan. White bar represents 5 μm.D. Magnification of the region in the dotted box to better show the colocalization of *Tb*MLP-myc.E. Western blot to show the specificity of the anti-HA antibody for p67 (lane 1: wild type, 2: trypanosomes transformed with *Tb*MLP-myc, 3: trypanosomes transformed with *Tb*MLP-myc and p67-HA). The lower panel shows Coomassie-stained gel to show equivalent loading.

The *TbMLP* transcript is constitutively expressed in both bloodstream and procyclic *T. brucei* (Supplementary Fig. S1), consistent with the observation that trypanosomes lack haem-oxygenase and both life-cycle stages require non-haem iron for survival and replication. To identify the subcellular location of the protein, we integrated an epitope tag into the endogenous gene (*Experimental procedures*). The final 933 bp of the *TbMLP* ORF was cloned in frame with 12 copies of a c-myc-derived epitope tag (Alsford and Horn, 2008). The tagged protein was localized by immunofluorescence with monoclonal antibody 9E10 directed against the c-myc epitope. This revealed that the protein was limited to vesicular structures occurring between the flagellar pocket/kinetoplast and the nucleus, suggestive of the endolysosomal compartment (Fig. 2A). The antibody recognized a single band only in the transformed cells and not in the wild type on a Western blot indicating the specificity (Fig. 2B, lane TbMLP-myc). As a marker for the lysosome, the p67 lysosomal glycoprotein gene was tagged with an influenza HA epitope by *in-situ* integration using the vector p2708 (Kelley *et al*., 1999; Kelly *et al*., 2007). In dual tagged cells, there was almost complete colocalization between *Tb*MLP and p67 (Fig. 2C and D, where D is a magnification of the boxed region in C) indicating that *Tb*MLP was located primarily in the lysosomal compartment of the endocytic pathway. Again, the antibody recognized a specific protein only in cells transformed with the p67-HA construct (Fig. 2E, lane 3).

### RNAi-mediated knock-down of TbMLP results in increased susceptibility to iron chelators

**Figure 3:**
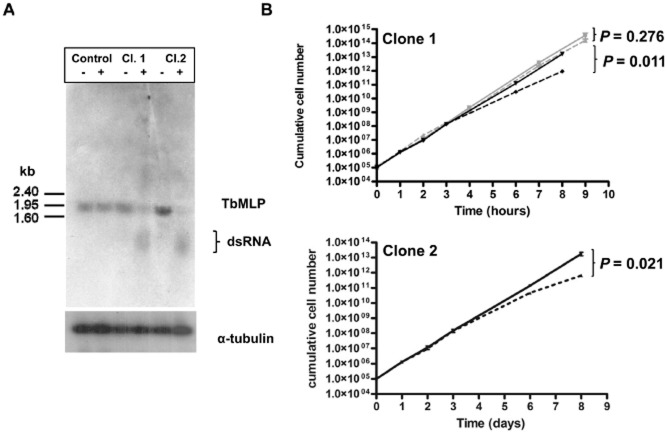
RNAi-mediated depletion of *Tb*MLP has a delayed effect on growth of bloodstream-form parasites.A. Northern blot showing loss of the *TbMLP* transcript on induction of RNAi. Cultures of the parental cell line (2T1) and two clones transformed with the RNAi construct were split in two. One flask of each had tetracycline added (1 μg ml^−1^, indicated by +). The other did not (indicated by −). After 24 h of induction, total RNA was extracted, blotted and hybridized with the *TbMLP* ORF. α-Tubulin mRNA is shown as loading control.B. Growth curves of RNAi clones in the presence (broken black line) or absence (solid black line) of tetracycline. Cells were seeded at 10^5^ ml^−1^ and counted after 24 h. They were then diluted back to 10^5^ ml^−1^ every 24 h to maintain exponential growth. For longer counting intervals, cells were diluted appropriately. Each clone was plated in triplicate and each well counted twice. *P-*values for the final time point are indicated to the right of the curves. The growth of the parental inducible cell line used to generate the RNAi transfectants is shown in grey (solid line without tetracycline, dashed line with tetracycline).

*Trypanosoma brucei* bloodstream-form cells were transformed with an inducible RNAi construct targeted against the *TbMLP* mRNA (*Experimental procedures*). Expression of the ‘hairpin’ transcript was induced by addition of tetracycline (1 μg ml^−1^). The ∼ 2 kb *TbMLP* mRNA was substantially depleted within 24 h (Fig. 3A). Quantification by phosphorimaging showed that the transcript had been knocked down between 70% (clone 1) and 85% (clone 2) (Fig. 3A). After three days, growth of the induced cells began to slow, in comparison with the uninduced parasites (Fig. 3B; black lines). To control for any effect of tetracycline, the growth of the parental cell line was also followed in the presence and absence of inducer (Fig. 3B; top panel, grey lines). There was no change in growth rates for the parental cells. The difference in growth following depletion of *TbMLP* mRNA was shown to be statistically significant (*P*-values for final time point indicated on Fig. 3B). There was no indication of cell death, or changes in morphology or motility.

**Figure 4:**
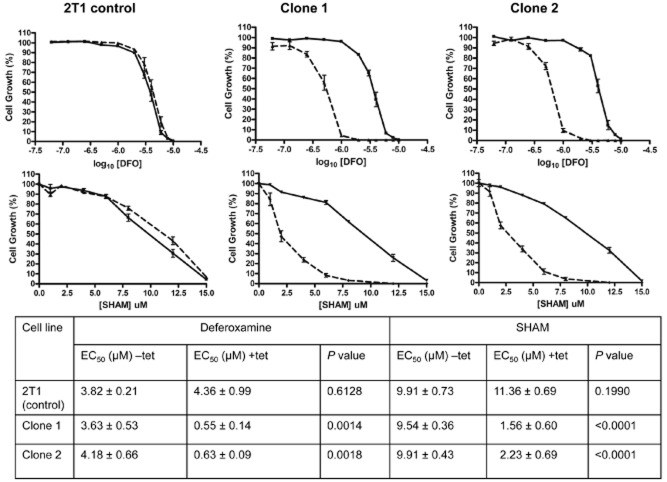
*Tb*MLP downregulation leads to enhanced susceptibility to iron chelators. Cells were induced for 24 h and then plated in varying concentrations of deferoxamine (DFO) or SHAM. Plates were incubated for 72 h and growth measured using alamarBlue™. The baseline was calculated using alamarBlue™ incubated with medium alone. Control cells (2T1 strain) are shown in the left hand panels and two independent RNAi clones in the centre and right hand panels. Solid line indicates cells incubated without tetracycline (−tet) and broken line indicates cells induced with tetracycline (+tet). EC_50_ values (±SEM) are illustrated in the table. EC_50_ values were determined using Graphpad PRISM software and the significance of the difference between EC_50_ values (+/−tet) was calculated using the F-test (*P*-values shown in table).

To investigate if *Tb*MLP is involved in iron acquisition, we tested the susceptibility of these trypanosomes to iron-chelators following induction of the RNAi response. We used two chelators, the bacterial siderophore deferoxamine, which is used clinically in the treatment of iron overload syndromes, and salicylhydroxamic acid (SHAM), which has been shown to specifically inhibit the trypanosome alternative oxidase, a mitochondrial di-iron protein essential to the bloodstream form (Evans and Brown, 1973; Clarkson *et al*., 1989; Chaudhuri *et al*., 2006). Cells were induced with tetracycline for 24 h, incubated with the chelators at a range of concentrations and grown for 3 days (*Experimental procedures*). Two RNAi clones (derived from separate transfections) examined in parallel were found to be approximately sevenfold more susceptible to SHAM and deferoxamine (Fig. 4), suggesting restricted iron availability when *Tb*MLP1 expression is reduced. The EC_50_ obtained for deferoxamine for the 2T1 controls was similar to that previously noted for *T. brucei* strain S427 bloodstream forms (Breidbach *et al*., 2002).

Since iron is potentially toxic, most organisms strictly control its uptake. In *T. brucei*, the transcripts for the transferrin receptor (*ESAG6/7*) are rapidly upregulated in response to iron deficiency (Fast *et al*., 1999). We investigated if *TbMLP* expression was also subject to upregulation. When trypanosomes were cultured in conditions known to induce accumulation of the *ESAG6/7* mRNA (25 μM deferoxamine for 24 h), the level of *ESAG6/7* mRNA increased by approximately eightfold (Supplementary Fig. S2A). However, the *TbMLP* mRNA levels remained constant following this treatment, suggesting that *Tb*MLP is unlikely to be responsive to the same regulatory mechanism as ESAG6/7. We also found that the effects of RNAi-mediated knock-down of *TbMLP* mRNA were insufficient to promote upregulation of the transferrin receptor mRNA (Supplementary Fig. S2B). These results could reflect that while deferoxamine treatment results in accumulation of Fe^3+^:deferoxamine complexes in the lysosome, downregulation of *Tb*MLP would tend to create an accumulation of ‘free’ Fe^2+^ since the cognate lysosomal reductase should still be active.

### The *TbMLP* gene can only be deleted in the presence of an expressed ectopic copy

**Figure 5:**
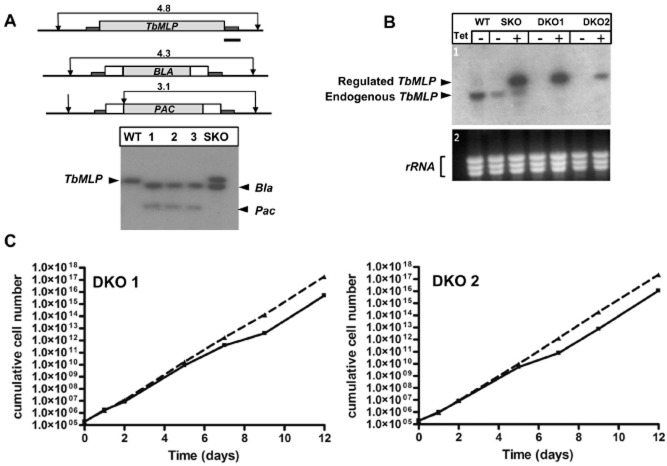
*TbMLP* can be deleted in the presence of a regulated ectopic copy.A. Deletion of endogenous *TbMLP* alleles. Map shows location of HindIII sites and the small black line indicates the probe. The Southern blot of genomic DNA digested with HindIII shows interruption of the two endogenous alleles in the conditional null mutants [lanes 1, 2 and 3 are individual clones, the wild type (WT) and the heterozygote (SKO) are shown for comparison].B. Repression of conditional *Tb*MLP expression leads to downregulation within 24 h. Total RNA was isolated and blotted from wild type (WT), heterozygote (SKO) and two conditional null clones (DKO1 and DKO2), +tet, cells incubated with tetracycline (1 μg ml^−1^), −tet, cells washed (× 3) and removed from tetracycline for 24 h.C. Growth phenotype of conditional null mutant recapitulates the RNAi phenotype. The broken black line in each case indicates the cumulative growth of the cell line maintained on tetracycline. The solid black line indicates the growth of the same line after removal of tetracycline by washing. A representative experiment is shown for each clone.

As induction of RNAi produced a relatively minor growth defect, we attempted to generate *TbMLP* null mutants to determine if there was redundancy in the mechanisms that trypanosomes use for iron acquisition. Targeting vectors were constructed to delete both copies of *TbMLP* and replace them with blasticidin (*BLA*) and puromycin (*PAC*) resistance cassettes. Replacement of the first allele was straightforward; however, we were unable to delete the second. On occasions where dual-resistant parasites were selected, we found that this had resulted from integration at alternative sites in the genome (data not shown). These outcomes are generally taken as being indicative of an essential gene. To confirm this, we generated a conditional null mutant by inserting a tetracycline-inducible ectopic copy of *TbMLP* into *TbMLP^+/−^* heterozygote parasites and then attempted to delete the second *TbMLP* allele. For this experiment, we utilized the *T. brucei* 2T1 cell line which contains constitutively expressed tetracycline repressor and a tagged rRNA locus suitable for inducible expression (Alsford *et al*., 2005; Alsford and Horn, 2008). The first *TbMLP* allele was deleted in the 2T1 cell line using the *BLA* construct. An inducible copy of *TbMLP* was then integrated into the tagged rRNA locus. Expression was shown to be inducible and repressible, with silencing of the transgene occurring within 24 h of tetracycline withdrawal (Fig. 5B; lane SKO). Finally, the second *TbMLP* allele was deleted, with transformants maintained on tetracycline to promote expression of the ectopic copy. Clones in which both endogenous alleles had been deleted were readily obtained (Fig. 5A; lanes 1, 2 and 3).

Following withdrawal of tetracycline from the cultured conditional null mutants, the *TbMLP* mRNA signal was virtually undetectable by 24 h (Fig. 5B; lanes DKO1 −tet and DKO2 −tet). Quantitative RT-PCR analysis of these RNA samples indicated a drop of between 2500- and 3500-fold in the transcript levels for *TbMLP* between the induced and repressed states (Supplementary Tables S1 and S2). However, we observed only a mild detrimental effect on growth, which became apparent after 4–6 days (Fig. 5C). This phenotype was soon masked by the outgrowth of cells which had returned to the normal growth rate. This suggests there may be an alternative mechanism available to complement for the loss of *TbMLP*. Analysis of one of the conditional cell lines which had returned to the normal growth rate revealed a recombination event that had deleted a copy of the *tet* repressor along with its phleomycin resistance cassette (data not shown). This clone had taken over the population since no phleomycin-resistant cells could be recovered. Reversion events such as this are commonly seen with conditional knockouts of essential genes in trypanosomes (Krieger *et al*., 2000).

**Figure 6:**
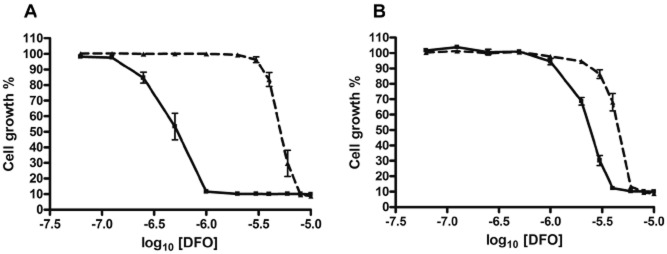
Conditional null mutants also display enhanced susceptibility to deferoxamine (DFO). Cells were repressed for 24 h and then plated in varying concentrations of deferoxamine. Control cells were maintained in tetracycline (1 μg ml^−1^). Plates were incubated for 72 h and growth measured using alamarBlue™ as in Fig. 4. The broken black lines represent growth in the presence of tetracycline. The solid black lines represent growth in the absence of tetracycline.A. DKO1 (EC_50_ +tet 5.11 ± 0.57 μM, −tet 0.48 ± 0.11 μM; *P* < 0.0002).B. DKO2. (EC_50_ +tet 4.35 ± 0.27 μM, −tet 2.28 ± 0.27 μM; *P* < 0.0016).EC_50_ values were calculated using Graphpad PRISM software and the significance of the difference between EC_50_ values (+/−tet for each clone) was calculated using the F-test.

The conditional null mutants were also investigated for susceptibility to iron chelators. As with the RNAi cells, the null mutants displayed increased sensitivity to the growth inhibitory effects of deferoxamine when *TbMLP* expression was downregulated. Of the two conditional null mutant clones tested, one was 11-fold more susceptible and the other twofold when tetracycline was withdrawn (Fig. 6). The variation between clones could be due to the presence of escape mutants/transcriptional leakiness. Nevertheless, it is clear that downregulation of *TbMLP* expression, either in an RNAi or in a conditional null mutant background, results in increased sensitivity to iron chelating agents. Deferoxamine has a high specificity for iron and the effects observed are unlikely to be due to chelation of other biologically important transition metal ions.

### Depletion of *Tb*MLP results in increased susceptibility to superoxide

One consequence of depletion in intracellular iron levels should be a concomitant decrease in the activities of iron-dependent enzymes. We had already seen an indication of this when the induction of RNAi against *TbMLP* resulted in an enhanced susceptibility to SHAM suggesting a decreased level of alternative oxidase activity. This enzyme is the primary target of SHAM (Evans and Brown, 1973; Opperdoes *et al*., 1976; Brohn and Clarkson, 1978; Clarkson *et al*., 1989; Helfert *et al*., 2001; Ott *et al*., 2006). To further test this, we subjected both the conditional null mutants and the RNAi cells to the superoxide generator paraquat (methyl viologen). Trypanosomes express only iron-dependent superoxide dismutases (Fe-SODs) (Dufernez *et al*., 2006; Wilkinson *et al*., 2006). RNAi mediated ablation of one of these (*TbSOD A*) resulted in enhanced susceptibility to paraquat in bloodstream-form *T. brucei* (Wilkinson *et al*., 2006). We reasoned, therefore, that if depletion of *Tb*MLP did result in a lower availability of intracellular iron, then the activity of *Tb*SOD A should be reduced and hence the cells should become more susceptible to paraquat.

**Figure 7:**
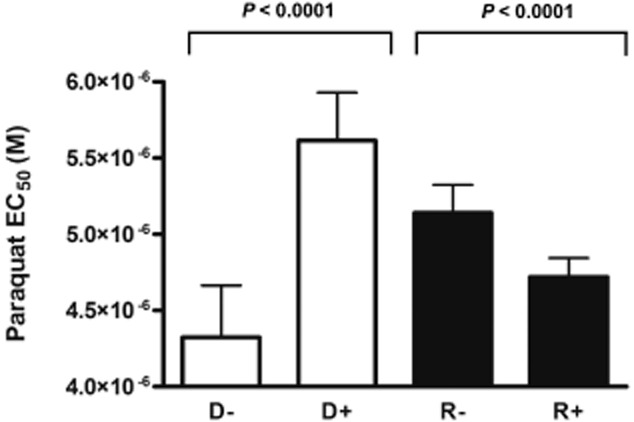
Parasites with reduced levels of *Tb*MLP exhibit enhanced susceptibility to superoxide. Expression of *Tb*MLP in bloodstream-form *T. brucei* was repressed (conditional null cells −tet, RNAi cells +tet) for 48 h and cells were then plated in varying concentrations of paraquat. Plates were incubated for 72 h and growth measured using alamarBlue™. EC_50_ values were calculated using variable slope sigmoidal curve fit (Graphpad PRISM software) and the significance of the difference between EC_50_ values (+/−tet for each clone) was calculated using the F-test (*P*-values indicated). Lanes D: DKO (EC_50_ −tet 4.33 ± 0.34 μM, +tet 5.62 ± 0.30 μM); lanes R: RNAi line (EC_50_ −tet 5.14 ± 0.18 μM, +tet 4.72 ± 0.12 μM)

Incubation of the conditional null mutants in the absence of tetracycline, which leads to downregulation of *TbMLP*, resulted in greater sensitivity to paraquat (Fig. 7). A similar trend was observed in the RNAi cell line. In this case, addition of tetracycline led to reduced expression and a concomitant increase in paraquat susceptibility (Fig. 7). The finding of similar results in both RNAi and conditional null mutants excludes the possibility of an antioxidant effect of tetracycline itself on paraquat susceptibility.

### Depletion of *Tb*MLP does not affect virulence *in vivo*

**Figure 8:**
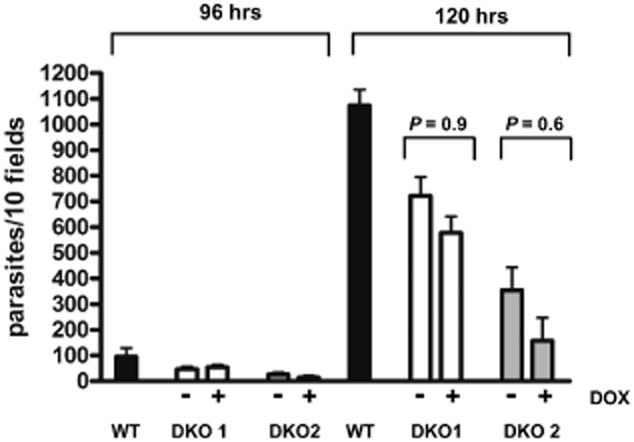
Conditional null mutants retain virulence *in vivo* in the absence of *Tb*MLP induction. Groups of five mice were infected with the null mutant cell lines DKO1 and DKO2 in the presence or absence of doxycycline (*Experimental procedures*). The course of infection was followed by tail-blood parasitaemia. The chart indicates the number of parasites observed in 10 fields of a Giemsa-stained blood smear. The data were analysed with Graphpad PRISM software. For both conditional null mutants there was no significant difference between the course of parasitaemia in the presence or absence of inducer (*P*-values shown above bars). All mice were euthanized after 5 days to prevent unnecessary suffering in accordance with statutory requirements.

To test whether the extent to which expression of *Tb*MLP is required for virulence, we infected female BALB/c mice with the conditional mutant. The parental strain, *T. brucei* S427, is monomorphic and causes an acute and rapidly lethal infection in mice. One group of five mice was given doxycycline (200 μg ml^−1^) in their drinking water to maintain expression of the transgene. The other group was maintained in the absence of the inducer, so that expression should be repressed. Mice were infected with only 500 trypanosomes to minimize the chance of an escape mutant being present in the initial inoculum. Trypanosomes in the repressed group were taken off tetracycline for 48 h prior to infection to ensure minimal expression of *TbMLP*. All mice showed patent parasitaemia by 3 days post infection and had to be culled due to high parasitaemia on day 5. Examination of blood smears revealed no significant differences in parasitaemia between the groups (Fig. 8).

## Discussion

Iron is an essential nutrient for almost all pathogenic microorganisms. Its role in human infectious trypanosomatids has been recently reviewed (Taylor and Kelly, 2010). African trypanosomes obtain iron by uptake of transferrin, the carrier protein that circulates in the host bloodstream. The fate of the endocytosed transferrin protein has been well documented; it is degraded in the parasite lysosome, and the proteolytic fragments are ejected back into the bloodstream (Steverding *et al*., 1995). However, the mechanism by which the released iron escapes from the lysosome into the parasite cytosol is less clear. Here, we have identified and characterized a *T. brucei* protein (*Tb*MLP) orthologous to the mucolipin family of ion channels that we propose as a candidate for this role. It is expressed in both bloodstream and insect stages of the parasite and is confined to the endocytic system, with the highest expression being found in the p67-positive compartment corresponding to the single terminal lysosome (Kelley *et al*., 1999).

Using an RNAi-based approach, we showed that although trypanosomes could initially divide normally when expression of *Tb*MLP had been reduced, growth retardation would occur after 3 days. This is in broad agreement with previous studies which suggested that trypanosomes have an internal iron store capable of supporting 48 h of cell division (approximately seven generations) in the absence of exogenous iron supplies (Steverding, 1998; van Luenen *et al*., 2005). In contrast to this relatively minor growth defect, knock-down of *TbMLP* did have a significant impact on the susceptibility of trypanosomes to the iron-chelators deferoxamine and SHAM. These compounds exhibit their effects in different parts of the cell. Deferoxamine is largely confined to the endosomal system as it cannot readily cross membranes (Kurz *et al*., 2006), while SHAM can penetrate to the mitochondrion where it specifically inhibits the di-iron protein trypanosome alternative oxidase (Evans and Brown, 1973; Clarkson *et al*., 1989; Ott *et al*., 2006). Thus, the effect seen with *TbMLP* RNAi suggests a general defect in the iron supply. This phenotype was confirmed in the conditional null mutant (eliminating the possibility of off-target RNAi effects being responsible). In addition, depletion of *TbMLP* by RNAi or in the conditional null mutants also resulted in a greater susceptibility to the superoxide generator paraquat. Since the SOD repertoire of trypanosomes is entirely dependent on iron for catalytic activity, this result provides further evidence that *Tb*MLP plays a significant role in the delivery of iron to the trypanosome cytoplasm for incorporation into metalloenzymes. The effect of *Tb*MLP depletion on paraquat sensitivity was relatively small, but reproducible. Two parameters may have a role in the level of sensitivity observed. The first is that SOD itself may have a long half-life. Therefore even when the iron supply is compromised, a reasonable level of activity can be maintained for some time, as has been demonstrated in trypanosomes treated with deferoxamine (Breidbach *et al*., 2002). Second, as iron itself plays a major role in the generation of oxidative stress [the superoxide-driven Fenton reaction (Halliwell and Gutteridge, 2007)], then manipulations which result in a depletion of intracellular labile iron will reduce the level of oxidative stress generated during paraquat administration. This could, to some extent, counterbalance the decrease in SOD activity.

Given the putative topology and the lysosomal location of the protein, our data therefore support a role for *Tb*MLP as the *T. brucei* endosomal iron-release channel. Unequivocal experimental evidence of transport activity is difficult to achieve in the absence of a heterologous expression system that mimics the trypanosome lysosomal membrane. Our data suggested that *Tb*MLP could be an essential protein since deletion of both alleles was only possible in the presence of an ectopic copy of the gene. However, conditional null mutants could grow *in vivo* and *in vitro* in the absence of detectable *TbMLP* transcripts. Either basal level expression of the transgene is sufficient to meet the needs of the parasite, or there is an alternative pathway for iron uptake. It was noticeable however that there appeared to be strong selection for adaptation, as back extrapolation of the outgrowth lines to the *x*-axis in Fig. 5C indicated that normally growing cells were appearing in the population within 2 days of the removal of tetracycline from the medium. One population had adapted by rearrangement of the tetracycline repressor genes that control ectopic expression of *Tb*MLP.

We did attempt to measure the labile iron pool in these trypanosomes using a calcein quenching assay (Esposito *et al*., 2002). The level detected in control cells was close to the limit of sensitivity of the technique (∼ 100 nM), rendering accurate measurement unachievable (Esposito *et al*., 2002; Kakhlon and Cabantchik, 2002).

It remains possible that the RNAi growth phenotype observed after 3 days could result from a reduced supply of metals other than iron (or a combination), since mammalian MCOLN1 is known to be permeable to Ca^2+^ and Zn^2+^ (Dong *et al*., 2008; 2010[Bibr b15],[Bibr b16]; Eichelsdoerfer *et al*., 2010). In trypanosomes however, the major players in Ca^2+^ homeostasis are known to be the plasma membrane, mitochondrion and acidocalcisomes, rather than the lysosome (Moreno and Docampo, 2003; Docampo and Lukes, 2012). Significantly, no effect on cell morphology was seen with either the RNAi cell lines or the conditional null mutants. Loss of function mutations in mammalian MCOLN 1 are known to cause gross defects in lysosomal morphology, endocytosis and autophagy. The lack of the serine lipase domain in *Tb*MLP may account for the lack of these phenotypes.

Whether *Tb*MLP is the only means by which iron is able to access the cytosol in *T. brucei* bloodstream forms remains to be resolved. However, the properties of this protein suggest a major role in the transfer of transition metal ions, notably Fe^2+^, across the lysosomal membrane and into the cytoplasm. Because parasites must obtain essential nutrients from their hosts, nutrient uptake mechanisms could prove to be an exploitable Achilles' heel for new therapies.

## Experimental procedures

### Parasite culture and genetic manipulation

*Trypanosoma brucei brucei* strain Lister 427 bloodstream trypomastigotes were cultured in HMI-9 (Invitrogen) supplemented with 10% v/v tetracycline-free fetal bovine serum (BioSera), penicillin/streptomycin (GibcoBRL) and β-mercaptoethanol (Sigma) at 37°C in a 5% CO_2_ atmosphere (Hirumi and Hirumi, 1989). *T. brucei* 2T1 cell lines carrying two copies of the *tet* repressor gene and a tagged inducible expression locus were cultured as above, but supplemented with phleomycin (1 μg ml^−1^) and puromycin (1 μg ml^−1^) (Alsford *et al*., 2005). RNAi cells were maintained on phleomycin (1 μg ml^−1^) and hygromycin (0.5 μg ml^−1^). For the conditional null mutants, cells were maintained on phleomycin (1 μg ml^−1^), puromycin (1 μg ml^−1^), blasticidin (10 μg ml^−1^), hygromycin (0.5 μg ml^−1^) and tetracycline (1 μg ml^−1^). Conditional null mutants were not maintained in culture for more than 2 weeks unless required by the experiment, to avoid selection of escape mutants. Growth curves and drug susceptibility assays were carried out in the absence of selection agents, except for tetracycline as required.

For transfection, parasites (5 × 10^7^) were pelleted and resuspended in 100 μl human T-cell nucleofection buffer (Lonza) and electroporated using program X.001 on the Nucleofector. The cells were resuspended in 200 ml HMI-9 and allowed to recover for 4–6 h. The appropriate selective agent was then added (at concentrations mentioned above) and the cells seeded in 48-well plates. Positive clones were isolated after 6-day selection at 37°C.

### Constructs

For tagging the endogenous *TbMLP* gene with a carboxyl-terminal c-myc tag, the 3′ 933 bp of *TbMLP* (Tb927.7.950 at http://tritrypdb.org) was amplified using primers:

1. F: 5′-aaaa*ggcgcgcc*ATTCCATGAGAAGGTCAGATGAC
2. R: 5′-cccc*tctaga*CCCCCTCAACTTCCCCAATATTT

(restriction sites in italics used for cloning). The product was digested with AscI and XbaI and ligated into AscI/XbaI-digested pNAT^x12MYC^ [a gift from Sam Alsford, LSHTM (Alsford and Horn, 2008)]. For transfection, the construct was linearized using the unique SphI site within the *TbMLP* ORF.

The RNAi construct was based on stem-loop vector pRPa^iSL^ (Alsford and Horn, 2008). A *TbMLP* gene-internal fragment of 407 bp from nt 661–1067, identified using RNAit software (http://trypanofan.path.cam.ac.uk/software/RNAit.html), was amplified using primers:

1. F: 5′-tttt*gggcccggtacc*GCAGTATCGAGGAGCGTTTC
2. R: 5′-aaaa*tctagaggatcc*AAAACCATCGACCACTACGC

The sense orientation was cloned by digestion with KpnI and BamHI, while the antisense was cloned with ApaI and XbaI, restriction sites indicated by italics.

### Targeted gene deletion and construction of conditional null mutant

Gene deletion constructs were generated by cloning the *TbMLP* 5′ and 3′ flanking sequences into drug resistance cassettes for blasticidin and puromycin. 356 bp of 5′-flanking DNA was amplified and cloned using primers:

1. F: 5′-aaaa*gcggccgc*GAATCATGATCAGCGAACCACG
2. R: 5′-tttt*ggatcc*ACAGCTTCGGATTCATATGTG

For the 3′-flanking DNA a 333 bp fragment was cloned using primers:

1. F: 5′-tttt*gggccc*GGTAGTTTCCTGCCCTTCTTAT
2. R: 5′-tttt*ggtacc*TGTTTCGACTAGGGTTCGCTGA.

The constructs were digested with NotI and KpnI for transfection.

A tetracycline regulated copy of the *TbMLP* gene was created by inserting the full-length ORF into the vector pRP^c6MYCn^ with a stop codon inserted (Alsford and Horn, 2008).

### Immunofluorescence

Exponentially growing parasites were fixed in 2% paraformaldehyde in normal growth medium. The cells were then pelleted and washed in PBS. Fixed cells were dotted onto slides and air-dried. For internal labelling, cells were permeabilized by incubation in 0.5% Triton X-100/PBS for 20 min. Slides were washed 3× in PBS then blocked for 15 min in 50% FBS/PBS. Primary antibody was added at an appropriate dilution in 20% FBS/PBS and the slides incubated for 1 h then washed 3× in PBS. Secondary antibody was added at an appropriate dilution in 20% FBS/PBS and the slides incubated for 45 min. Slides were washed 3× in PBS, then mounted in 1:1 PBS:glycerol with DAPI. Slides were examined on a Zeiss Axioplan 510 confocal laser scanning microscope.

### RNA interference/conditional null mutant drug susceptibility assays

For RNAi cell lines, one flask was induced with tetracycline (1 μg ml^−1^) while the other remained uninduced, as a control. For conditional nulls, the cells were maintained on tetracycline (1 μg ml^−1^) and prior to the experiment were washed 3× in 1 volume of medium without tetracycline (estimated final concentration of tetracycline < 1 pg ml^−1^). The population was then split in two and tetracycline added back to the control population. After 24 h of induction/repression, the cells were seeded into 96-well plates at 10^4^ ml^−1^. For paraquat assays the induction/repression was prolonged to 48 h prior to drug testing to allow for depletion of Fe-SOD activity mediated by loss of *Tb*MLP-dependent iron transport. The appropriate drug concentration was added to each well and the plates incubated at 37°C for 2 days. Twenty microlitres of alamarBlue™ was then added to each well and the plates incubated at 37°C overnight. Fluorescence was read in a Gemini Fluorimeter at λ_ex_ 530 nm and λ_em_ 585 nm with a cut-off set at 570 nm (Molecular Devices).

### cDNA synthesis and qPCR quantification of MLP mRNA levels in conditional null mutant

cDNA was synthesized using the Superscript VILO cDNA synthesis kit (Invitrogen). For each RNA sample, three separate cDNA syntheses were carried out. Briefly, 1 μg total RNA was reverse transcribed for 2 h at 42°C. The reaction was terminated at 85°C for 5 min. Each was taken forward into the qPCR reaction. cDNA equivalent to 100 ng was amplified using primers:

1. MLP QF 5′-ACATACCGACTGCAGCAACTGG and
2. MLP QR 5′-CCTAGTACATCGCTTGCTTGA, giving a product of 169 bp
3. TERT QF 5′-GAGCGTGTGACTTCCGAAGG and
4. TERT QR 5′-AGGAACTGTCACGGAGTTTGC, giving a product of 108 bp.

The reaction was carried out using the QuantiTect® SYBR® Green kit (Qiagen) in a Rotor-gene 3000 instrument (Corbett Research). Cycling conditions were: 1 cycle of 95°C for 15 min, followed by 40 cycles of 94°C for 15 s, 58°C for 20 s and 72°C for 30 s, Data acquisition was carried out after each 72°C stage and the products were analysed by a melt curve after the final cycle.

The reference transcript for normalization was TbTERT (telomerase reverse transcriptase), as this had previously been validated in a variety of conditions (Brenndorfer and Boshart, 2010). A standard curve was derived by amplification of a series of 10-fold dilutions of the target PCR products.

For RNAi, knock-down was quantified by phosphorimager analysis of Northern blots probed with the *Tb*MLP ORF (test probe) and β-tubulin (internal control for normalization of loading). Analysis was performed using a Typhoon imager and ImageQuant software (GE Healthcare Life Sciences). Phosphorimager analysis was used rather than qPCR to ensure that only full-length mRNA was quantified.

### *In vivo* infection

Conditional null mutants maintained on tetracycline, were washed 3× in HMI-9 and split into two flasks. One flask had tetracycline (1 μg ml^−1^) added back. The flasks were incubated for 48 h to ensure repression of the transgene. Female BALB/c mice were infected with 500 bloodstream trypomastigotes i.v. Five mice were infected in each group. Mice which received the tetracycline induced cells had doxycycline (200 μg ml^−1^) in their drinking water with 5% sucrose. The other mice were given 5% sucrose. Doxycycline treatment was started 24 h prior to infection. Parasitaemia was monitored by tail bleeds and mice were euthanized when a high parasitaemia (non-recoverable) was apparent, in accordance with statutory animal welfare requirements and UK Home Office regulations.
