# Supplementary material for: Evidence that transport of iron from the lysosome to the cytosol in African trypanosomes is mediated by a mucolipin orthologue
Source: Mol Microbiol. 2013 Jun 27;89(3):420–32. doi: 10.1111/mmi.12285 (PMC3828870; doi:10.1111/mmi.12285)
Supplement: Supplementary file 1 [file mmi0089-0420-sd1.pdf]

Supplementary Information

Table S1: C_t data for qPCR analysis of conditional null mutant lines.

Clone	Condition	Replicate	*MLP* qPCR (C_t) mean (n=3)	*MLP* Standard Deviation	*TERT* qPCR (C_t) mean (n=3)	*TERT* Standard Deviation
1	- tet (silenced)	a	34.39	0.3	17.41	0.02
		b	34.43	0.71	17.67	0.57
		c	35	1.09	17.3	0.37
1	+tet (induced)	a	22.48	0.1	16.79	0.47
		b	22.45	0.08	18	0.41
		c	23.54	0.83	17.54	0.63
2	- tet (silenced)	a	35.03	0.49	16.13	1.55
		b	35.17	1.16	18.33	0.85
		c	34.43	0.32	18.21	0.51
2	+ tet (induced)	a	24.71	0.39	18.26	0.5
		b	24.28	0.54	18.57	0.56
		c	24.71	0.19	18.73	0.2

Columns labelled MLP refer to amplification of the test product derived from *TbMLP*, *TERT* refers to the calibration product derived from telomerase reverse transcriptase. Each replicate cDNA sample was subject to 3 separate amplifications. C_t data are shown as the mean of the 3 with standard deviation.

Table S2: $\Delta\Delta C_t$ data for qPCR analysis of conditional null mutant cell lines: Calculated using the mean of the 3 replicates shown in table S1.

Clone	ΔC_t induced	ΔC_t silenced	$\Delta\Delta C_t$ (induced-silenced)	Fold change
1	5.38	17.15	-11.77	~3500
2	6.05	17.32	-11.27	~2500

Supplementary Figure Legends

Fig. S1. Expression of *TbMLP* mRNA is constitutive. Total RNA (10 µg) from cultured bloodstream form (BS) and procyclic (PC) trypanosomes was fractionated on a 1.2 % agarose gel. The blot was hybridised with the *TbMLP* ORF. An ethidium stained photograph of the ribosomal RNA bands prior to blotting is shown to indicate loading.

Fig. S2. A. TbMLP is not co-regulated with the transferrin receptor (TfR). Wild type trypanosomes were incubated for 24 hours in the absence (control lane) or presence of 25 µM deferoxamine (DFO lane). RNA was isolated and electrophoresed. The blot was probed with the indicated genes. An α-tubulin probe was used as a loading control. **B.** RNAi-mediated depletion of TbMLP does not result in up-regulation of TfR expression at the RNA level. TbMLP RNAi cells were induced for 96 hours and RNA was isolated and blotted. In the presence of tetracycline (lane +) the *TbMLP* transcript was not undetectable, however the *TfR* mRNA level was unchanged in contrast to the effect of deferoxamine.

Fig.S1

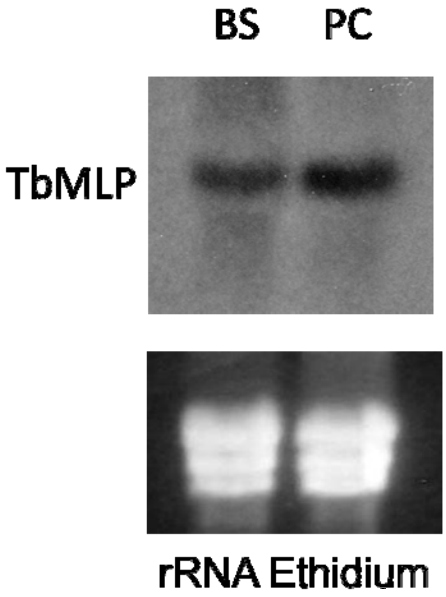

Fig.S2

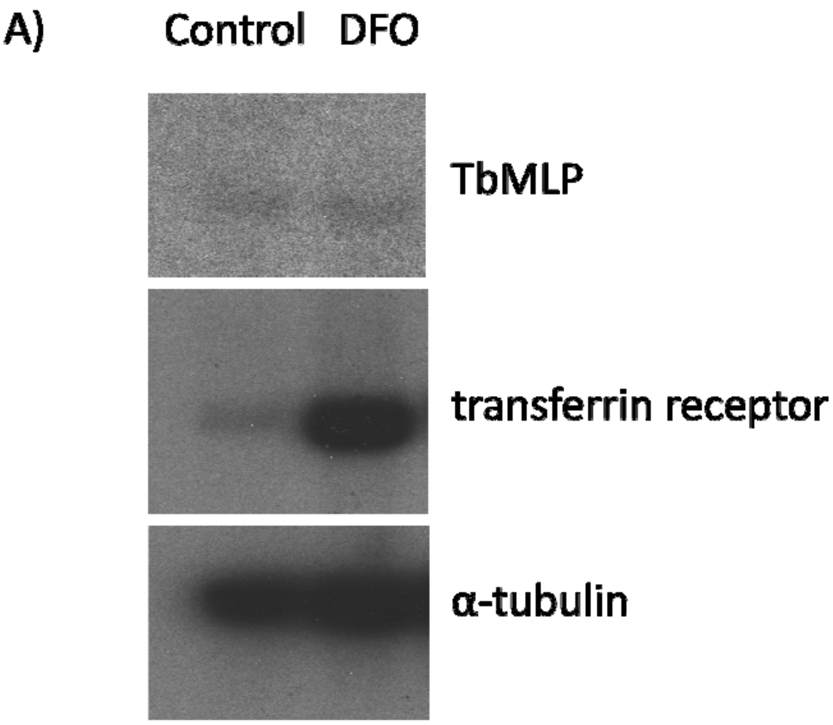
A)
Control
DFO
TbMLP
transferrin receptor
α-tubulin

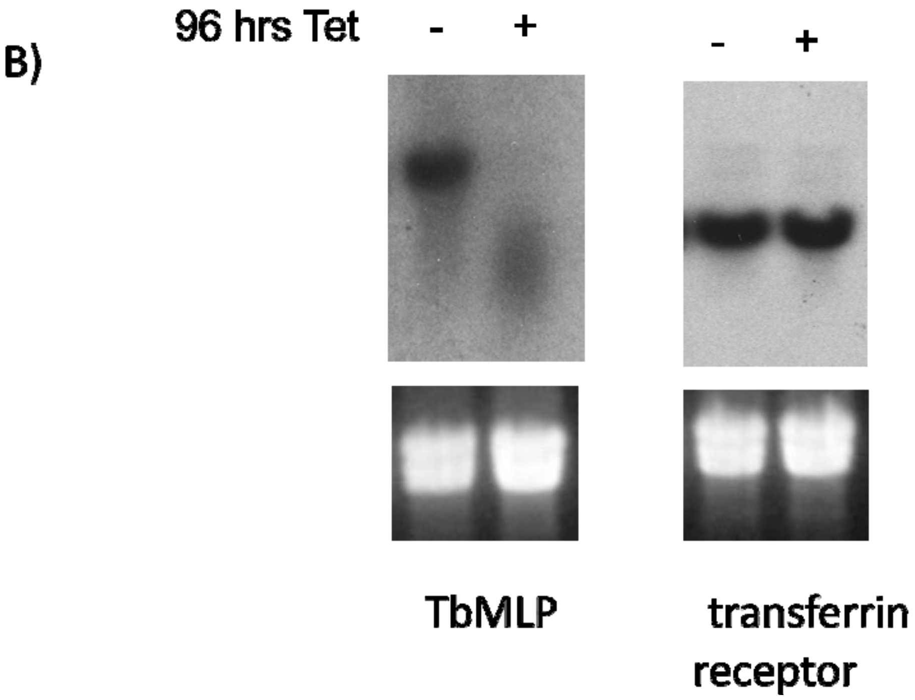
B)
96 hrs Tet
-
+
-
+
TbMLP
transferrin receptor
